# A Doppler-Tolerant Ultrasonic Multiple Access Localization System for Human Gait Analysis

**DOI:** 10.3390/s18082447

**Published:** 2018-07-27

**Authors:** Karalikkadan Ashhar, Mohammad Omar Khyam, Cheong Boon Soh, Keng He Kong

**Affiliations:** 1 School of Electrical and Electronic Engineering, Nanyang Technological University, Singapore 639798, Singapore; ecbsoh@ntu.edu.sg; 2 Department of Mechanical Engineering, Virginia Tech, Blacksburg, VA 24060, USA; mok@vt.edu; 3 Department of Rehabilitation Medicine, Tan Tock Seng Hospital, 17 Ang Mo Kio Ave 9, Singapore 569766, Singapore; keng_he_kong@ttsh.com.sg

**Keywords:** motion tracking, channel multiple access, ultrasonic localization, gait analysis, doppler shift compensation

## Abstract

Ranging based on ultrasonic sensors can be used for tracking wearable mobile nodes accurately for a long duration and can be a cost-effective method for human movement analysis in rehabilitation clinics. In this paper, we present a Doppler-tolerant ultrasonic multiple access localization system to analyze gait parameters in human subjects. We employ multiple access methods using linear chirp wave-forms and narrow-band piezoelectric transducers. A Doppler shift compensation Technique is also incorporated without compromising on the tracking accuracy. The system developed was used for tracking the trajectory of both lower limbs of five healthy adults during a treadmill walk. An optical motion capture system was used as the reference to compare the performance. The average Root Mean Square Error values between the 3D coordinates estimated from the proposed system and the reference system while tracking both lower limbs during treadmill walk experiment by 5 subjects were found to be 16.75, 14.68 and 20.20 mm respectively along X, Y and Z-directions. Errors in the estimation of spatial and temporal parameters from the proposed system were also quantified. These promising results show that narrowband ultrasonic sensors can be utilized to accurately track more than one mobile node for human gait analysis.

## 1. Introduction

Time of flight-based ultrasonic positioning systems can be cost-effective alternatives for long-term motion tracking in rehabilitation clinics and gait analysis [1]. The gold standard for clinical gait analysis is the optical motion capture system with multiple high-speed cameras [2] which is costly, sensitive to variations in the light intensity, clutter and shadow. It also requires dedicated laboratories, experienced operators and complex calibration procedures [1]. Motion capture systems such as Vicon MX—with more than 6 cameras which provide sub-millimeter accuracy cost tens of thousand dollars [3]. Motion tracking systems employing micro-electromechanical sensors such as accelerometers and gyroscopes cannot be used for reliable long-term tracking due to pronounced drift [4]. Systems employing more than one type of sensors to complement the performance of each other such as the systems explained in [4,5,6,7] increased the overall cost and complexity of the system. Ultrawideband (UWB) systems can also be used for time of flight-based localization. However, accurate clock synchronization between the mobile nodes is difficult due to the high propagation speed of the UWB waves. The cost of the system also increases as the receivers need an analogue to digital converters which can sample signals with high bandwidth [1].

Ultrasonic waves can be utilized for accurate small-scale localization. The system becomes cheap and wearable with low power consumption if narrowband piezoelectric sensors are utilized. Such a small scale localization circuit can be employed for long-term human gait analysis in rehabilitation clinics and in unrestrained environments [1]. In [1], single tone pulses were used for localizing a single mobile node. A frequency modulated signal such as a linear chirp can provide narrower cross-correlation peak at the receiver side than that obtained by a single frequency burst [8]. It can also enable the system for scaling up the number of mobile nodes that can be tracked simultaneously by exploiting various modulation techniques such as Orthogonal Frequency Division Multiplexing (OFDM) [9] or other chirp diversity multiple access methods [10].

Broadband ultrasonic sensors were utilized to implement Direct Sequence Spread Spectrum (DSSS) [11], Frequency Hopping Spread Spectrum (FHSS) [12] and Code Division Multiple Access (CDMA) [13] for localization in some of the previous papers. Broadband sensors can provide better noise tolerance, update rate and channel multiple access. However, the hardware cost increased significantly and higher voltages and computing requirements made the system unfit for wearable applications. Also, the accuracy while locating fast moving targets using broadband ultrasonic signals is degraded by the Doppler effect [14]. Polyvinylidene fluoride (PVDF) [15], Electromechanical film (EMFi) [16] and Capacitive/Piezoelectric micro-machined ultrasonic transducers (cMUTs/pMUTs) [17] can be used as broadband ultrasonic transducers for indoor localization. However, PVDF, EMFi and cMUTs require a high voltage supply to provide enough transmission power which makes these systems unfit for mobile tracking applications. Also, the mechanical setup needed in case of PVDF due to the length variations of films makes it difficult to use in custom tracking applications [18]. pMUTs suffer from a divergence of the transmitted beam [17]. In [19], a resistor and an inductance were added in series with the narrowband ultrasonic sensor to increase the bandwidth of the transmitter, but additional transmitted power loss will be introduced by these components. Therefore, in this work, we investigate the effective utilization of the bandwidth of narrowband ultrasonic sensors for our particular application.

The distances of the mobile nodes whose coordinates, are to be estimated from three or more anchor nodes with known coordinates, are required for localization. These distances are calculated from the time the ultrasonic waves takes to travel from mobile nodes to anchor nodes or vice versa. For human motion tracking, we prefer active mobile node architecture—that is, the ultrasonic emitters are moving targets attached to body parts. In a passive mobile node architecture, it will be difficult to collect and store the data in a central system. The distance travelled by the wave can be estimated from time of flight, *t* by Equation (1) [20].

(1) d=(331.5+0.6×T)×t

where *T* represents the room temperature in degree centigrade. Let the coordinates of the mobile node be (x,y,z) and that of the *i*th anchor node be $\left(x_{i},y_{i},z_{i}\right)$, then the estimate of range, $d_{i}$ can be expressed as

(2) $$d_{i}={\left(x_{i}-x\right)}^{2}+{\left(y_{i}-y\right)}^{2}+{\left(z_{i}-z\right)}^{2}+n_{i}$$

where, $n_{i}$ is the measurement noise and i=1,2,…,m where m≥3. Equation (2) can be solved for (x,y,z) using some iterative algorithms such as least squares estimation [21], Kalman filter [1] or a particle filter [22].

Various methods can be used to accurately find the time at which the transmitted waves reach the receiver. Some of them are: a simple thresholding technique, cross-correlation of the transmitted signal with the received signal and finding the peak or by measuring the phase shift between transmitted and received signals. The cross-correlation-based method provides accurate results and is the standard method for Time of Flight estimation [23]. Even though the accuracy of the phase shift method is higher than the cross-correlation based method, the maximum range that can be measured with this method alone is limited to one wavelength [24]. A combination of the phase shift method and cross-correlation method was explained in [24] to improve the maximum range to about 6 m. However, the noise in the cross-correlation results should be less than one wavelength which might not be true in a typical indoor environment. A threshold-based phase-correlation method was explained in [25], however, this method can only provide improved performance if the signal to noise ratio is high and there are no multiple access interference. Cross-correlation accuracy improves when the waveform is a frequency modulated one such as a linear chirp [26]. This also aids channel multiple access. In this work, we implement a cross-correlation method with linear chirp waves for ultrasonic ranging. In practice, the transmitted ultrasonic waves can travel by more than one path to reach the receiver in a cluttered environment leading to multi-path interference. An earliest peak search method can remove most of the multi-path components in real life implementations [27].

Moving ultrasonic transmitters produces a Doppler shift in the received frequency and it affects the performance of the system. The Doppler compensation techniques such as correlator bank receivers, which is the most common method lead to high processing time and resources and provide less accuracy [28]. We propose a method with simple narrowband linear chirp signals which can compensate the range-Doppler coupling in linear chirp signals.

The rest of the paper is organized as follows: In Section 2, the mathematical model of the multiple access and multi-path interference problem in ultrasonic ranging is explained. Section 3 provides the solution for multiple access and Doppler shift using linearly varying chirp signals. Experimental procedures for multiple access performance and gait analysis are demonstrated in Section 4. The results from experiments are discussed in Section 5 and Section 6 concludes the work with suggestions for future work.

## 2. Description of Problem

Let the number of mobile nodes transmitting ultrasonic waves simultaneously be *N*. The signal transmitted by each mobile can be represented by $S_{i}$ where *i* = 1, 2, …, *N*. The signal received by one receiver in the absence of multi-path effect can be represented as [26]:

(3) $$R\left(t\right)=\sum_{i=1}^{N}A_{i}\times \left(h_{i}*S_{i}\right)\left(t-t_{i}\right)+n\left(t\right)$$

where, $A_{i}$ and $t_{i}$ are the amplitude and time of flight of the signal received from *i*th transmitter. $h_{t}$, $n_{t}$ and ∗ represent channel impulse response, additive white Gaussian noise (AWGN) and the convolution operator. However, Equation (3) takes into account only the direct signal path. In an indoor environment, multiple delayed and attenuated replicas of the transmitted signals are also received due to the reflections from nearby surfaces. The received signal from *i*th transmitter can be modeled as shown in Equation (4).

(4) $$A_{i}\times \left(h_{i}*S_{i}\right)\left(t\right)\approx \sum_{m=1}^{M}A_{im}\times S_{i}\left(t-t_{m}\right)$$

where, *M* is the number of multi-path components received for *i*th signal and $t_{m}=t_{i}+\Delta t_{im}$ where $\Delta t_{im}=0$ for the direct path without reflections. After reception, R(t) is correlated with a locally stored copy of emitted signal, $S_{j}$. The waveform after correlation with *k*th waveform can be represented as [26]:

(5) $$\begin{array}{c} C_{k}\left(t\right)=\left(\sum_{i=1}^{N}A_{i}\times \left(h_{i}*S_{i}\right)\left(t-t_{i}\right)+n\left(t\right)\right)★S_{k}\left(t\right)\\ =\left(\sum_{m=1}^{M}A_{km}\times S_{k}\left(t-\left(t_{k}+\Delta t_{km}\right)\right)\right)★S_{k}\left(t\right)\\ +\left(\sum_{i\neq k}\sum_{m=1}^{M}A_{im}\times S_{i}\left(t-\left(t_{i}+\Delta t_{im}\right)\right)+n\left(t\right)\right)★S_{k}\left(t\right) \end{array}$$

In Equation (5), the first term on the right hand side is the auto correlation of transmitted signal distorted by the multi-path interference. The distance information from *k*th transmitter is embedded in $t_{k}$. The second term on the right hand side represents the multiple access interference and the channel noise. Index of the peak value of $C_{k}\left(t\right)$ can be used to estimate $t_{k}$.

Equation (5) is applicable for stationary transmitter and receiver. However, if the transmitter is moving relative to the receivers, the frequency of the received signal, $S_{i}$ is shifted up or down proportional to the velocity due to the Doppler shift. The errors introduced by the Doppler effect in the range measurement depends on the signal design. We use chirp signals with linearly varying frequency. Let $S_{i}\left(t\right)$ represents a linear chirp signal with the frequency varying from $f_{1}$ to $f_{2}$ in time $T_{s}$. Let the relative velocity between the ultrasonic transmitter and the receiver be $v_{r}$ with transmitter moving away from the receiver. Then the expansion or compression factor of the received signal, *b* can be obtained as shown in Equation (6).

(6) $$b=\frac{c}{c+v_{r}}$$

where, *c* is the velocity of ultrasound in air. The received signal after Doppler shift will be a chirp signal with the frequency varying from $bf_{1}$ to $bf_{2}$ in time $T_{s}/b$ [29]. The estimated time delay, *t* in the received signal will be a linear function of the frequency [30].

(7) $$t=t_{0}-\frac{T_{s}}{\left(f_{2}-f_{1}\right)}\times f$$

Let the frequency shift due to Doppler shift be Δf and error in estimated time be Δt. Then,

(8) $$\Delta t=\frac{-T_{s}}{\left(f_{2}-f_{1}\right)}\times \Delta f$$

which is a function of the velocity of moving target for a particular chirp signal.

## 3. Proposed Method

Various methods to reduce multiple access interference using chirp signals are explained in [26]. In our experiments, two mobile nodes are attached to the left and right lower limbs of the subject whose gait patterns are to be analyzed. In [31], a similar gait analysis model was explained. However, spatial division multiple access was utilized with two separate boards with four fixed anchor nodes on either side of the body and both mobile nodes were simultaneously transmitting. Here, the two boards need to be synchronized and the positions of all the sensors need to be known without error. The limited transmission angle was taken advantage here for multiple access. To avoid errors due to Non-Line of Sight (NLOS), we need to increase the transmission angle of the sensors and this makes spatial division multiple access difficult. This is because the range estimation is prone to interference as multiple mobile nodes are transmitting the same waveform, which also affects the scalability of the system for more than two mobile nodes. In this paper, we test two methods for multiple access along with a Doppler compensation technique for moving targets. The multiple access performance with more than two narrowband ultrasonic sensors with 40 kHz center frequency transmitting simultaneously was explained in [10]. However, the performance when the targets are moving was not studied. In this work, we are finding the multiple access performance of two ultrasonic mobile nodes in static condition as well as in dynamic condition when the system is used for bilateral gait analysis.

### 3.1. Signal Design

In this work, we test two designs for the transmitted signal to minimize multiple access interference. In the first design, the rate of variation of chirp frequency is used to identify the mobile node from the transmitted signal [10]. That is one signal’s frequency linearly increases at the same time, the frequency of the other signal linearly decreases. In the second design, the orthogonality of the discrete frequency components of a chirp waveform was utilized for positioning two transducers simultaneously [10]. In the second design, the length of the signal is doubled and thus the computational cost also increases as the number of mobile nodes increase.

#### 3.1.1. Up and Down Chirp for Multiple Access

A linear chirp can be mathematically represented as:

(9) $$S_{i}\left(t\right)=rect\left(\frac{t}{T_{s}}\right)\times exp\left\{j2\pi \left(f_{si}t+\frac{1}{2}u_{i}t^{2}\right)+\phi \right\}$$

**Figure 1:**
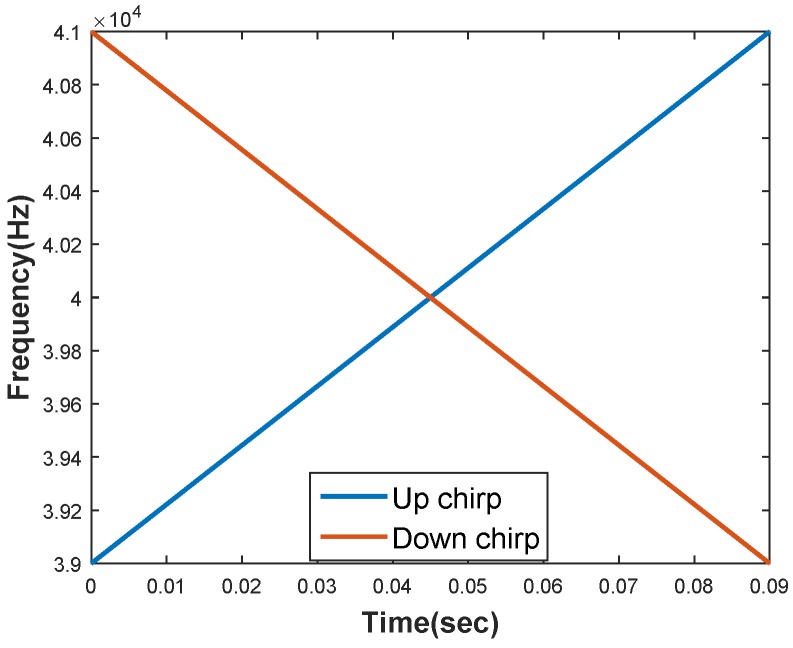
Illustration of up and down chirp rates for two signals with 39–41 kHz/9 ms and 41–39 kHz/9 ms chirp signals.

Here, rect(.) represents a rectangular window function. $T_{s}$ is the chirp signal duration. $f_{si}$ is the starting frequency, ϕ is the initial phase and is set to zero. $u_{i}=\left(f_{ei}-f_{si}\right)/T_{s}$ where $f_{si}$ and $f_{ei}$ are starting and ending frequencies respectively. The two signals required for our system are generated utilizing the additional degree of freedom for modulation compared with single tone signals. That is the chirp rate [32]. Let $S_{1}$ and $S_{2}$ be the signals (i=1,2), then for $S_{1}$, $f_{s1}=39\;\mathrm{kHz},f_{e1}=41\;\mathrm{kHz},u_{1}>0$ and for $S_{2}$, $f_{s2}=41\;\mathrm{kHz},f_{e2}=39\;\mathrm{kHz},u_{2}<0$ An illustration of the chirp signals used is shown in Figure 1.

#### 3.1.2. Orthogonal Frequency Division Multiple Access

In the second method, the orthogonality of discrete frequency components of the signals are utilized. Here we are using orthogonal chirp signals. Since we are having only two set of ultrasonic transmitters transmitting simultaneously, we used a simple technique to generate the signals. In time domain, the first up chirp signal generated in Equation (9) is concatenated in time domain with itself to get the first waveform. The second waveform is obtained by concatenating in time domain the negative of the up chirp signal with itself. Let Tx1 and Tx2 represent the two orthogonal signals to be generated and $S_{1}\left(t\right)$ represents the up chirp signal with signal duration $T_{s}/2$. Then,

(10) $$\begin{array}{cc} Tx1= & rect\left(\frac{t}{T_{s}/2}\right)S_{1}\left(t\right)+rect\left(\frac{t-T_{s}/2}{T_{s}/2}\right)S_{1}\left(t-T_{s}/2\right)\\ Tx2= & rect\left(\frac{t}{T_{s}/2}\right)S_{1}\left(t\right)-rect\left(\frac{t-T_{s}/2}{T_{s}/2}\right)S_{1}\left(t-T_{s}/2\right) \end{array}$$

**Figure 2:**
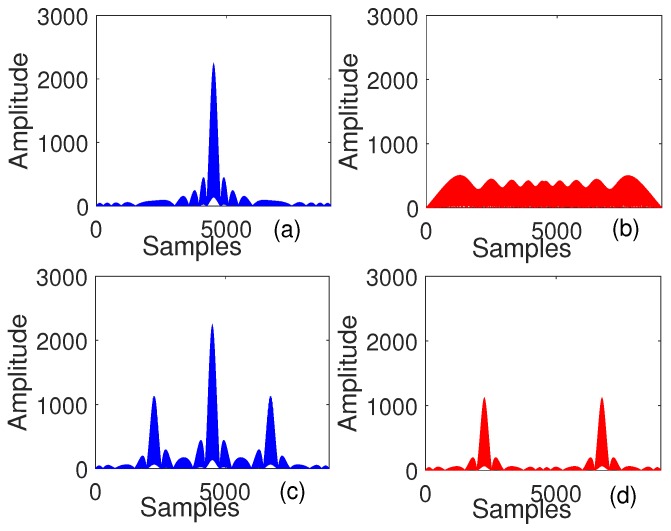
Comparison of (**a**) auto-correlation and (**b**) cross-correlation of $S_{1}$ and $S_{2}$ for up and down chirp signals and (**c**)auto correlation and (**d**) cross-correlation of Tx1 and Tx2 for orthogonal chirp signals.

In frequency domain, this operation corresponds to zero interleaving Discrete Fourier Transform (DFT) of $S_{1}$ to obtain frequency domain equivalent of Tx1 and shifting the frequency domain equivalent of Tx1 by one position to the right to get the frequency domain equivalent of Tx2 [9]. Although both signals, Tx1 and Tx2 are transmitted simultaneously from different transmitters, both signals are received by receiver without any interference as the multiplication of the frequency spectra of both results in zero. The auto correlation of $S_{1}$, cross-correlation of $S_{1}$ and $S_{2}$, auto correlation of Tx1 and cross-correlation of Tx1 and Tx2 and are provided in Figure 2.

### 3.2. Doppler Shift Compensation

Moving targets emitting ultrasonic waves lead to Doppler shift in the frequency of the signal received at the stationary receivers. This leads to large errors in the range values estimated. If orthogonal chirp signals are used as explained in Section 3.1.2, the Doppler shift can cause the signals to lose orthogonality and the multiple access cannot be possible, especially in this case as the bandwidth is limited. Linear frequency modulated signals are highly tolerant to Doppler effect [33]. In the case of linear chirp signals, the multiple access is rarely affected by the Doppler effect, but an error is introduced in the range measurement which is proportional to the velocity of moving target known as range-Doppler coupling [30]. This error will be positive or negative depending on whether we are using up or down chirp. We propose the use of linear chirp signals with positive and negative chirp rates of same magnitude to compensate for range-Doppler coupling. The range value estimated from the up-chirp and down-chirp can be represented as shown in Equation (11) [30].

(11) $$\begin{array}{cc} r1= & r0+\dot{r}\times T_{d}\;for\;up-chirp\\ r2= & r0-\dot{r}\times T_{d}\;for\;down-chirp \end{array}$$

where, r0 represents the actual range, r1 and r2 represents the estimated range by sending up- and down-chirp signals and $\dot{r}$ represents the target velocity in the ranging direction. $T_{d}$ is given by Equation (12).

(12) $$T_{d}=f0\times T_{s}/B$$

where, f0 is the centre frequency, $T_{s}$ is the signal duration and *B* is the swept bandwidth of the chirp signal. The mean value of r1 and r2 gives an estimate of r0.

As an example, Let f0=40 kHz, $T_{s}=7$ ms and B=2 kHz, Then $T_{d}=0.14$ s. Let us assume that the target is moving at a velocity, $\dot{r}=1$ m/s away from the receiver and the actual range, r0=1 m, then, r1=1.14 m and r2=0.86 m. The mean value of r1 and r2 gives us the actual range.

For gait analysis with ultrasonic transmitters attached to the lower limbs of the subject, the velocity of the lower limbs can be a few meters per second and the range-Doppler coupling causes errors which cannot be tolerated for narrowband signals with the 40 kHz centre frequency. Hence, we used two ranging cycles one with up chirp, (39–41 kHz)/7 ms and one with down chirp, (41–39 kHz)/7 ms. Each ranging takes 20 ms each and mean value is taken as one measurement. Thus the total data update rate of our system is 25 Hz.

## 4. Experimental Procedure

Each mobile node consists of one STM32F4-Discovery development board from ST Microelectronics. The chirp waveform to be transmitted is stored inside the flash memory of STM32F407VGT6 microcontroller at the core of the development board. This board has Direct Memory Access (DMA) controllers which can enable high performance and ultra-fast data transfer to the Digital to Analog Converter (DAC). We used sine chirp waveform from DAC as input to the driver circuit which consists of SN74AS04 hex inverters and IC-SN754410 which can boost the transmission power of the DAC output and can provide a bidirectional current drive of up to 1 A. The driver circuit was similar to the circuit explained in [34]. MURATA MA40S4S and MA40S4R were used as ultrasonic transmitters and receivers respectively. These are narrowband ultrasonic sensors which have a resonant frequency at 40 kHz and −6 dB bandwidth of about 2 kHz. The typical directivity of these sensors is 80 degrees. Both the IC-SN754410 and the STM32F4 control board were powered by an 11.1 V lithium-polymer battery and a 5 V rectifier. At the receiver side, the signal from the ultrasonic receiver was passed through a band-pass filter and amplifier. The received signal was sent through a second order band-pass filter and an amplifier. The lower and upper cut off frequencies of the band-pass filter were approximately 33 and 48 kHz. An inverting op-amp amplifier with a voltage gain of 11 was used. IC-LMV-358 was employed to design the band-pass filter and the amplifier. NI-USB 6343 data acquisition (DAQ) board was used to capture the signal which is connected to a PC through USB connection. The cross-correlation and further analysis were performed in Matlab. The trigger signal from the data acquisition board is transmitted to the mobile node through the STM32F4 board and the wireless transceiver. This acts as the clock synchronization signal between the ultrasonic transmitter and the receiver. The automatic acknowledgement and re-transmission features of the wireless transceivers were disabled to avoid any timing delays or jitters in the trigger signal.

### 4.1. Channel Multiple Access

**Figure 3:**
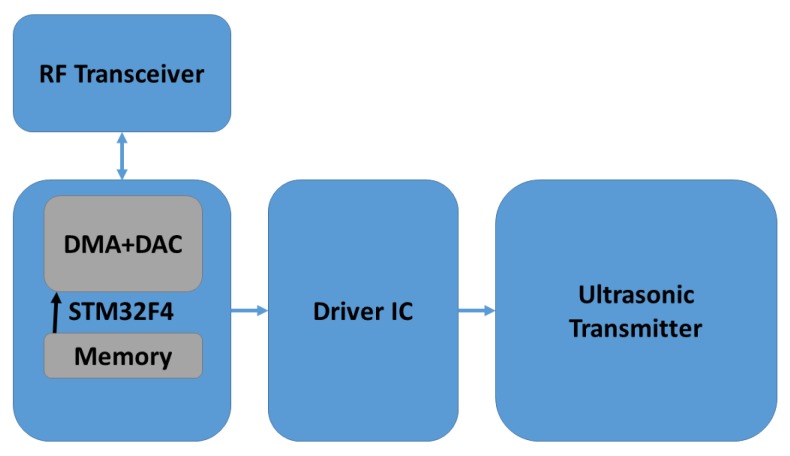
Block diagram of ultrasonic transmitter circuit (Mobile node).

**Figure 4:**
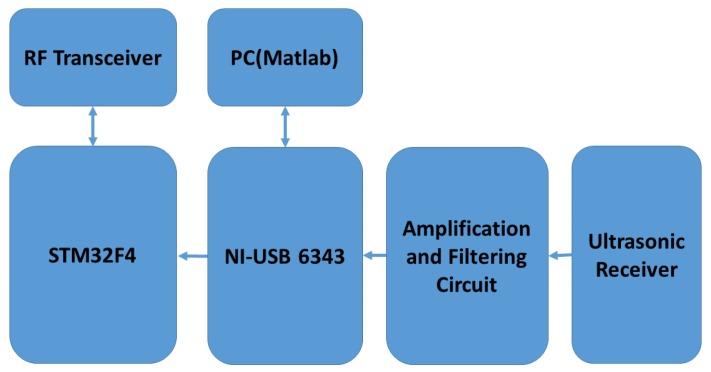
Block diagram of ultrasonic receiver circuit.

**Figure 5:**
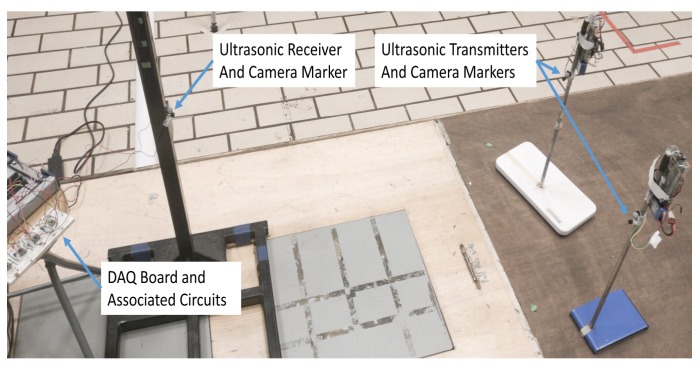
The experimental setup to study the multiple access performance.

We investigated the performance of the ranging system under multiple access interference with two transmitters in static conditions emitting ultrasonic waves simultaneously. The experimental setup included two transmitters and one receiver. For both signals explained in Section 3.1, measurements were taken for ten different locations of the two transmitters with respect to the receiver. Here the clock synchronization between the transmitters and the receiver was done using NRF24L01 Radio frequency (RF) modules. Block diagrams of the ultrasonic transmitter and receiver circuits are as shown in Figure 3 and Figure 4 respectively. Each measurement cycle was initiated by the DAQ board by starting to receive from the analogue input channel and simultaneously sending the trigger signal to all the mobile nodes through wireless radio frequency connection. The respective chirp signals stored in the memory of the mobile nodes are sent through the ultrasonic transmitters as soon as the trigger signal is received by the mobile nodes. At the anchor nodes, the received ultrasonic signal is a combination of transmitted waveform shifted by the corresponding propagation delays, the multiple access interference and channel noise. The environment in which the experiments were conducted was found to be of less multi-path interference. Cross-correlation with the corresponding transmitted waveform and finding the index of the earliest peak value of the envelope of cross-correlation, we obtain the propagation delay of the ultrasonic signals from each mobile node. The distance between the mobile node and the anchor node is obtained by substituting the value of propagation delay in Equation (1). The room temperature was assumed as constant and 23 ${}^{\circ }$C in all our experiments. Figure 5 shows the experimental setup used for studying the multiple access performance. The clock synchronization was implemented by a 2.4 GHz wireless connection between the mobile nodes and the anchor node.

### 4.2. Gait Tracking with the Proposed System

**Figure 6:**
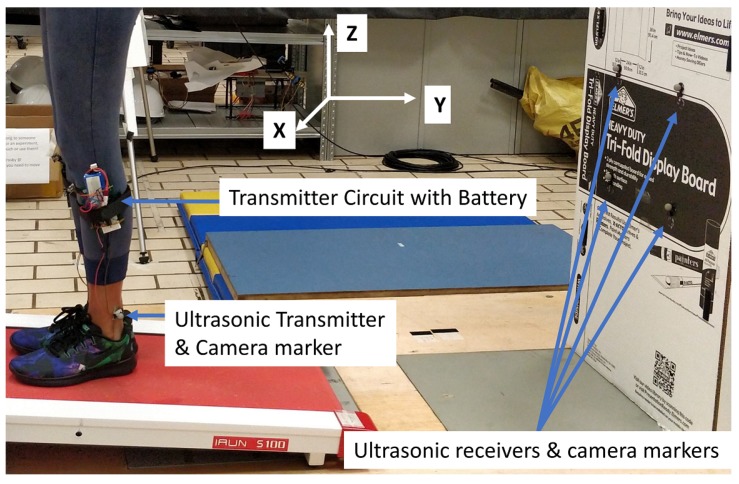
The setup for gait tracking with sensors attached to both lower limbs of the subject.

**Figure 7:**
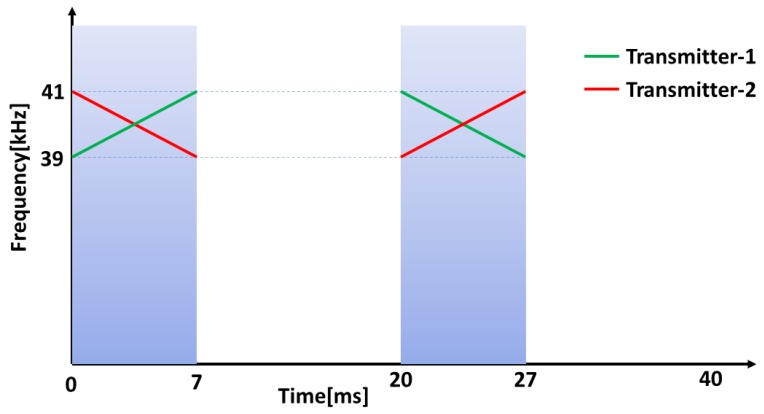
Representation of signals transmitted in one sampling instance of 40 ms.

A gait analysis system was proposed with the multiple access localization system by attaching the mobile nodes to each foot of the human subjects. The performance of the proposed gait analysis system was tested in an indoor environment with the subject walking on a treadmill. Two mobile nodes with ultrasonic transmitters were attached to either foot of the subject and four anchor nodes were placed at fixed positions with known coordinates behind the subject as shown in Figure 6. Ultrasonic sensors with battery and camera markers were attached to each lower limb of the subject with Velcro tape and double sided tape so that the camera marker and ultrasonic transducers are positioned just above the ankle joint as shown in Figure 6. The anchor nodes were placed at the corners of a 200 × 250 mm square and hence the 3D coordinates of the anchor nodes were set to (0, 0, 0), (200, 0, 0), (200, 0, 250) and (0, 0, 250). The four anchor nodes were placed about 600 mm above the ground to avoid one leg to interfere the Line of Sight(LOS) of the other during gait. In the 3D coordinate system, the walking direction was set as −Y, vertical direction as Z and lateral direction as X. The two mobile nodes were programmed to transmit at the same time using the multiple access technique explained in Section 3.1.1. One mobile node was made to transmit an up-chirp, (39–41 kHz)/7 ms at the rising edge of the clock signal and a down-chirp, (41–39 kHz)/7 ms at the falling edge of the clock signal. The second mobile node was made to transmit down-chirp at the rising edge of the clock signal and up-chirp at the falling edge of the clock signal. One instance of range measurement is as shown in Figure 7.

We neglected the slight changes in the velocity at which the up- and down-chirps are transmitted which are 20 ms apart. The clock signal which was of frequency 25 Hz was transmitted through wireless radio frequency connection from the DAQ to each mobile nodes. The command to start the measurements was given from the PC which was connected to the anchor nodes. Since we were using four analogue input channels from the DAQ board, the effective sample rate in each channel was set to 125,000 samples/s. The signals from four ultrasonic receivers were passed through band-pass filters and amplifiers before sending to the PC through a serial connection. The time taken by the waves to reach each of the four anchor nodes are estimated by cross-correlation of the received wave with the originally transmitted waveform. The mean value of time estimated from up and down-chirps was taken to remove the Doppler effect. The distances from each mobile nodes to four anchor nodes were calculated using Equation (1) at 23 ${}^{\circ }$C. All the distance measurements were passed through a median filter of order 3 to remove some NLOS errors and samples which lie outside the set upper and lower limits were removed. From eight distance measurements thus obtained, an Unscented Kalman Filter (UKF) was implemented to estimate the 3D coordinates of both the mobile nodes attached to each foot of the subject. As the experiments were conducted in low multi-path environment The earliest peak which is not less than 0.8 times the maximum peak was found to provide good results for multi-path effect compensation.

Five healthy subjects (4 Males and 1 Female) with age (25.6 ± 0.55) were recruited for the experiment. The experiments were conducted at the Motion Analysis Laboratory, School of Mechanical and Aerospace Engineering, Nanyang Technological University (NTU), Singapore. All the subjects involved in the experiment had a normal gait without any disorders which affect gait. Each subject was asked to walk on a treadmill at fixed walking speeds of 0.28, 0.56 and 0.83 m/s each for a duration of 1 min each with about 1-min break in between the trials. The Ultrasonic and Motion capture systems were started at the same time and slight time difference between the systems was offset during post-processing by finding the point at which the cross-correlation between the signals reaches a maximum value.

#### Unscented Kalman Filter

The state space model for estimating three dimensional coordinates from the measured distances can be represented by Equation (13). UKF [35] was used along with a smoothing filter to estimate the 3D coordinates of each mobile node from four distance measurements. In UKF, unscented transforms are used where, mean and co-variance of the target distribution are approximated directly instead of trying to approximate the non-linear function [35].

(13) $$\begin{array}{c} x_{k}={\mathrm{Ax}}_{k-1}+q_{k-1}\\ y_{k}=g\left(x_{k}\right)+r_{k} \end{array}$$

where $x_{k}$ denotes the state of the system and $y_{k}$ denotes the distances measured at *k*th moment, A, $g(x_{k})$, $q_{k}$ and $r_{k}$ are the state transition matrix, matrix of measured distances, process noise and the measurement noise respectively. The state of the system at the instant k is given as:

(14) $$x_{k}={\left[\begin{array}{cccccc} x & y & z & \dot{x} & \dot{y} & \dot{z} \end{array}\right]}^{T}$$

where *x*, *y* and *z* are the Cartesian coordinates of the mobile node to be tracked and $\dot{x},\dot{y}$ and $\dot{z}$ are the corresponding velocities.

(15) $$A=\left[\begin{array}{cc} I & T\times I\\ 0 & I \end{array}\right]$$

where T represents the sampling time and typical value used in our system is equal to 40 ms. $q_{k-1}∼N\left(0,Q_{k-1}\right)$ where $Q_{k-1}$ is the process noise co-variance matrix.

(16) $$Q_{k-1}=\left[\begin{array}{cc} \frac{T^{3}}{3}q^{2} & \frac{T^{2}}{2}q^{2}\\ \frac{T^{2}}{2}q^{2} & Tq^{2} \end{array}\right]$$

where *q* is a 3×3 diagonal matrix with diagonal elements $q_{x},q_{y}$ and $q_{z}$ which can be approximated as the standard deviations of velocity noise along *x*, *y* and *z* directions.

(17) $$g_{i}\left(x_{k}\right)=\sqrt{{\left(x_{k}-x_{i}\right)}^{2}+{\left(y_{k}-y_{i}\right)}^{2}+{\left(z_{k}-z_{i}\right)}^{2}}$$

where $x_{i},y_{i}$ and $z_{i}$ represents the coordinates of the fixed anchor nodes. $r_{i}∼N\left(0,R_{i}\right)$ where $R_{i}$ is the measurement noise co-variance matrix. $R_{i}$ can be approximated as a diagonal matrix with constant diagonal elements as the noise in each sensor measurement can be approximated as independent. $R_{i}=diag\left(e^{2},e^{2},e^{2},e^{2}\right)$ where, *e* can be estimated as the standard deviation of ultrasonic measurements which was calculated as 11 millimeters from experiments. The values of $q_{x},\;q_{y}$ and $q_{z}$ were empirically found out by minimizing the RMSE between the ultrasonic and camera data. Typical values used in our system were 500, 1000 and 700 respectively. An Unscented Raunch-Tung Striebel smoother was also used to smooth out the waveform [36]. The steps followed by UKF are briefly expalined here:

(A) Prediction
  - Generate the matrix of sigma points ($\chi_{k-1}$) and weights ($W_{i}^{mean},W_{i}^{cov}$) for unscented transformation. Here *n* is the number of states and n=6 and λ=−3.
    (18) $$\begin{array}{cc} \chi_{k-1}= & \left[m_{k-1}\;...\;m_{k-1}\right]+\\ & \sqrt{n+\lambda }\times \left[0\;\sqrt{P_{k-1}}\;-\sqrt{P_{k-1}}\right]\\ W_{0}^{mean}= & \frac{\lambda }{n+\lambda },\;W_{0}^{cov}=W_{0}^{mean}\\ W_{i}^{mean}= & W_{i}^{cov}=\frac{1}{2(n+\lambda )}\;\mathrm{for}\;i=1,\ldots ,2n \end{array}$$
  - Transmit the sigma points through the dynamic model.
    (19) $${\hat{\chi }}_{k,i}=f\left(\chi_{k-1,i}\right)\;i=1,\ldots ,2n+1\;$$
  - Predict the mean state matrix $m_{k}^{-}$ and co-variance matrix $P_{k}^{-}$ using weight matrices $W_{i-1}$.
    (20) $$\begin{array}{c} m_{k}^{-}=\sum_{i}W_{i-1}^{mean}{\hat{\chi }}_{k,i}\\ P_{k}^{-}=\sum_{i}W_{i-1}^{cov}\left({\hat{\chi }}_{k,i}-m_{k}^{-}\right){\left({\hat{\chi }}_{k,i}-m_{k}^{-}\right)}^{T}+Q_{k-1} \end{array}$$
(B) Update
  - Generate the matrix of sigma points.
    (21) $$\begin{array}{cc} \chi_{k}^{-}= & \left[m_{k}^{-}\;\ldots \;m_{k}^{-}\right]\\ & +\sqrt{n+\lambda }\times \left[0\;\sqrt{P_{k}^{-}}\;-\sqrt{P_{k}^{-}}\right] \end{array}$$
  - Transmit the sigma points through the dynamic model.
    (22) $${\hat{Y}}_{k,i}=h\left(\chi_{k,i}^{-}\right)$$
  - Compute the mean $\mu_{k}$, co-variance of the measurement $S_{k}$ and cross co-variance of the sate and measurement $C_{k}$ using weight matrices $W_{i}$.
    (23) $$\begin{array}{c} \mu_{k}=\sum_{i}W_{i}^{mean}{\hat{Y}}_{k,i}\\ S_{k}=\sum_{i}W_{i}^{cov}\left({\hat{Y}}_{k,i}-\mu_{k}\right){\left({\hat{Y}}_{k,i}-\mu_{k}\right)}^{T}+R_{k}\\ C_{k}=\sum_{i}W_{i}^{cov}\left(\chi_{k,i}^{-}-m_{k}^{-}\right){\left({\hat{Y}}_{k,i}-\mu_{k}\right)}^{T} \end{array}$$
  - Compute the Kalman gain *K*, state mean $m_{k}$ and co-variance $P_{k}$ after obtaining measurement update $y_{k}$ and update mean state and co-variance.
    (24) $$\begin{array}{c} K=C_{k}S_{k}^{-1}\\ m_{k}=m_{k}^{-}+K\left(y_{k}-\mu_{k}\right)\\ P_{k}=P_{k}^{-}-KS_{k}K^{T} \end{array}$$

### 4.3. Unscented Raunch-Tung Striebel Smoother (URTS)

Smoothing can be defined as the Bayesian statistical methodology that provides estimates of past state history of a time-varying system based on the history of noisy measurements obtained [36]. An additive form of URTS was implemented. The recursive algorithm starts from the last step and proceeds backwards and is explained in Appendix A.

## 5. Results and Discussions

We conducted experiments to find the best-case ranging accuracy for one transmitter and one receiver followed by an experiment with two transmitters and one receiver. For moving targets, various factors need to be taken care of such as the Doppler shift, the transmission patterns of the ultrasound and the angle between the axis of transmitter and receiver, which depend on the applications. Hence, we tested our system with healthy subjects walking on a treadmill.

### 5.1. Calibration of Reference System

Experiments were conducted at Motion Analysis lab (Motion Analysis Eagle System, Santa Rosa, CA, USA) in School of Mechanical and Aerospace Engineering, NTU, Singapore. This motion capture system consists of six high-speed cameras and cortex software which can provide sub-millimeter accuracy for motion tracking. System calibration was performed for static as well as dynamic conditions with a 4-point L-frame and a 3-point T-frame respectively. The sampling rate was set to 120 Hz. The accuracy of the reference system after calibration was found to be 0.364±0.192 mm (Mean ± Standard Deviation).

### 5.2. Performance in Environments with Multiple Access Interference

Initially, we evaluated the best-case ranging accuracy with one transmitter and one receiver in the absence of multiple access interference and with wireless clock synchronization between the transmitter and receiver. Here, both the transmitter and the receiver were in static condition. Eight different range values were measured. The constant offset value was removed and the error compared with the camera system was found to be −8.51±6.77 mm. The constant offset in the measurements can be due to various reasons such as the time taken by RF signal to reach the anchor nodes, the frequency variations due to the non-linear impulse response of the ultrasonic transceivers which leads to received signal not matching perfectly with the transmitted wave and thus the peak of the correlation might be shifted by a constant offset. To study the performance with multiple access interference, we employed the experimental setup explained in Section 4.1 with two static transmitters and one static receiver. One transmitters was made to transmit linearly varying up chirp and the other was made to transmit down chirp of length 9 ms (39–41 kHz/9 ms and 41–39 kHz/9 ms) at the same time. Measurements were taken for static conditions at different positions of mobile nodes with respect to one anchor node. Ten continuous measurements were taken every 100 ms for each of the ten different positions of the two mobile nodes. As there was a constant offset in the measurements, we compared the change in the measured distance value with the camera reference system. We subtracted the first set of distance measurements from the rest. The error in range values estimated from the ultrasonic and motion capture system was found to be 10.54±10.66 mm and 11.83±13.49 mm (Mean ± Standard Deviation) for first and the second mobile node respectively. A similar experiment was conducted for orthogonal signals explained in Section 3.1.2 with the length of the waveform same as that in the former experiment. Here the length of $S_{1}$ was set to 4.5 ms and Tx1 and Tx2 were of length 9 ms each. The errors in range measurements were found to be 2.16±23.54 mm and −4.80±25.40 mm respectively. Hence, both the multiple access methods were found to provide high accuracy for tracking two mobile nodes. The orthogonal chirp signals provided a lower mean error, while the variance of the error values was higher than that of up and down chirp.

### 5.3. Performance of Doppler Shift Compensation

**Figure 8:**
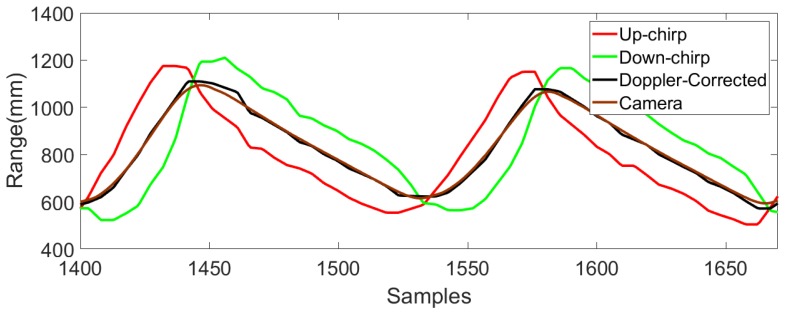
The comparison of the range measurements from one of the lower limbs to a fixed anchor node extracted from the proposed ultrasonic system using up-chirp, down-chirp, a mean of up and down-chirp and motion capture system for a walking speed of 0.83 m/s.

In this section, initially we examine whether Doppler shift has a dominating effect on tracking performance during walking or not and then find the effectiveness of our Doppler compensation technique. Initially we find the instantaneous velocity at which the foot moves away from the anchor node plane when the treadmill is operated at 0.83 m/s and theoretically find the maximum error that may affect the measurements. We selected one subject’s optical motion tracker gait data to find the instantaneous velocity of one of the lower limbs. The component of instantaneous velocity along the direction of the line connecting the center of the four anchor nodes and the moving mobile node was estimated. The maximum and the minimum of the velocity of the foot was found to be 1.3 and −0.7 m/s. Using Equation (11), the maximum and minimum error that is introduced in the measurement with either up-chirp or down-chirp was found to be 182 mm and −98 mm respectively which is 31.38% and 16.9% of maximum movement along Y-direction which is 580 mm. This error is not acceptable and we need proper Doppler compensation techniques for accurate measurements. To evaluate the effectiveness of the proposed Doppler shift compensation further, although we could use a pendulum model, we did not. The reason is twofold. Firstly, our gait analysis results in Figure 8 and Table 1 clearly show the effectiveness of the proposed Doppler shift compensation algorithm. Secondly, a pendulum model is generally used to evaluate the tracking performance as it follows a particular (known) trajectory [37]. However, we employed a sophisticated optical setup to determine the walking trajectory with a precision of 0.36±0.19 mm. In fact, we believe that our setup is more realistic than a pendulum setup. The distances between the mobile nodes and four anchor nodes when one subject walks at 0.83 m/s were obtained from the proposed system with Doppler correction and without Doppler correction using either up-chirp or a down-chirp. The mean absolute error (MAE) was calculated to compare the accuracy in each case and is listed in Table 1. The results of ultrasonic ranging before and after Doppler shift compensation presented in Figure 8 and Table 1 not only show that Doppler shift was dominating during walking but also the effectiveness of the proposed Doppler shift compensation algorithm in such an environment.

### 5.4. Performance of Gait Tracking with Proposed Method

**Figure 9:**
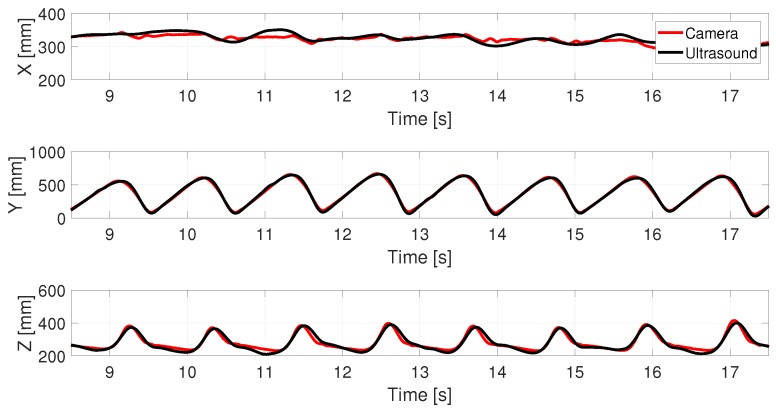
The comparison of the 3D coordinates of one marker attached to one of the lower limbs extracted from the proposed ultrasonic system and motion capture system for a walking speed of 0.83 m/s.

We implemented the multiple access method with up and down chirp signals along with the Doppler compensation explained in Section 3.2 in a low multi-path environment. Multi-path interference was less in the environment where the experiment was conducted and thus no compensation was required. Root Mean Square Error (RMSE) between the 3D trajectories obtained from the proposed ultrasonic system and the reference camera-based system was used as a metric to benchmark the system performance. RMSE for each foot for five subjects is shown in Table 2. The first few samples before the subject starts walking were removed while calculating RMSE. The comparison of the 3D coordinates extracted from the proposed and reference systems are as shown in Figure 9.

The average values of RMSE along *x*, *y* and *z* directions were found to be 16.75, 14.68 and 20.20 mm respectively. The results show that the system can provide high accuracy tracking along with good multiple access performance. The Pearson’s Correlation Coefficient (PCC) between the trajectories were calculated in the Y and Z directions. The mean PCC values were found to be 0.995 and 0.996 along Y (horizontal) direction and 0.899 and 0.9326 along Z (vertical) direction respectively for left and right foot.

#### Extraction of Spatial and Temporal Parameters

The step length and cycle time/stride time were extracted from the trajectories. The step length was defined as the difference between the maximum and the minimum of Y-coordinate for one stride and the cycle time/stride time was defined as the time between adjacent minima in one stride [1]. The mean and standard deviation of the error between the parameters obtained from both the systems at low and high walking speeds are listed in Table 3 and Table 4. It can be observed from the tables that the errors in step length and stride time are comparable to the results for one mobile node in [38] and slightly higher at high walking speeds. A Wilcoxon rank sum test was conducted to test the null hypothesis that the gait parameters extracted from both the systems after removing the mean value of linear trend follow a distribution with equal medians for all the subjects at all speeds for each foot. All the tests failed to reject the null hypothesis with p greater than 0.5 at 95% confidence level.

**Figure 10:**
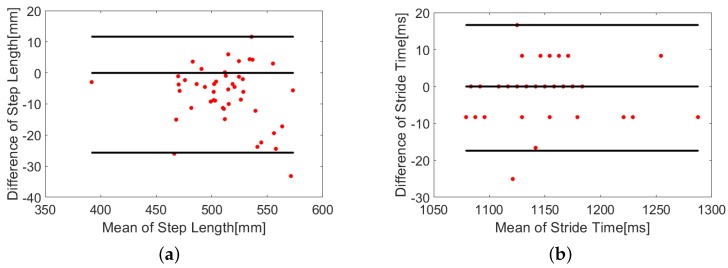
Bland-Altman plots comparing (**a**) step lengths and (**b**) stride time calculated from the proposed system with the motion capture system at 0.83 m/s walking speed for one foot of a subject. The upper and lower limits are set to mean difference ± 2× standard deviation (SD).

The Bland-Altman plots [39] depicting the difference between the gait parameters extracted from both systems are shown in Figure 10. The upper and lower limits of agreement were mean difference ± 2× standard deviation (SD) of the difference. The plots show an agreement between both the systems with small limits of agreement. The least count for representation of temporal parameters in the plots was 8 ms as the sampling rate of the reference system was 120 Hz, which was found to be about 0.6% of the average stride time and can be neglected.

## 6. Conclusions and Future Work

In this paper, the design of a gait analysis system to track both the lower limbs simultaneously during an indoor treadmill walk was proposed with a Doppler-tolerant multiple access ultrasonic localization system. We utilized various chirp signals as pulse compression with chirp signals can provide high ranging accuracy. The accuracy of the gait trajectories obtained from the proposed system was benchmarked with an optical motion capture system for 3 different walking speeds and five subjects. The system provided high accuracy for tracking along all three coordinate axes for both the mobile nodes at 0.28, 0.56 and 0.83 m/s. The average values of RMSE along *x*, *y* and *z* directions were found to be 16.75, 14.68 and 20.20 mm respectively. A high correlation between the trajectories was observed along horizontal and vertical directions. The errors in the spatial and temporal parameters estimated from the proposed system compared with the motion capture system were also quantified.

In future, we will be using redundant anchor nodes and omnidirectional ultrasonic transducers to remove NLOS errors due to moving body parts. The size, weight and power consumption of the wireless mobile nodes also need to be taken care of in future work. The sensors can be designed in the form of wearable bands to the upper and lower limbs. To scale the number of mobile nodes, the orthogonality of various chirp rates and other chirp waveform need to be studied in detail. The range resolution of a sensor is defined as the minimum separation of two targets transmitting the same waveform that can be resolved as separate targets. The range resolution was found to be about 172 mm in our system. If the multi-path range lies within 172 mm of the LOS range, there might not be two separate peaks and the resultant peak might be shifted from the original value, we will also be addressing this problem in our future work. Sensors with higher bandwidth provide higher resolution and better signal power at the receiver for chirp signals. The development of custom-made ultrasonic transducers makes the system robust and provides options for future developments. In the future, we plan to recruit subjects with various neurological disorders to find how this system can be effectively used for daily clinical gait analysis in the rehabilitation process.

The main disadvantages of the proposed gait analysis system are the errors caused due to the non-line of sight and the limited directivity of the ultrasonic transducers. Loose clothes of the subject which may obstruct the LOS can lead to errors in the measurement. In this work, we did not try to optimize the size of the wearable sensors and the power consumption. These issues will be addressed in future work.

## Figures and Tables

**Table 1:** Comparison of MAE for range measurement with and without Doppler correction.

Transmitter No.	Range to Anchor No.	MAE Using Up-Chirp (mm)	MAE Using Down-Chirp (mm)	MAE after Doppler Correction (mm)
1	1	103.72	99.03	9.45
	2	105.45	100.31	10.85
	3	94.95	85.88	11.92
	4	93.99	84.34	12.16
2	1	269.90	109.16	10.88
	2	270.88	116.09	10.95
	3	241.58	97.61	13.13
	4	241.93	95.78	16.43

**Table 2:** RMSE between the proposed and the reference system.

Subject	Axis	0.28 m/s		0.56 m/s		0.83 m/s	
		L	R	L	R	L	R
1	X	11.77	13.42	10.66	12.28	14.73	18.77
	Y	9.02	12.92	12.56	11.30	16.07	17.59
	Z	13.84	17.81	14.72	11.87	21.37	19.17
2	X	8.17	25.50	12.83	7.99	15.63	8.60
	Y	11.97	18.26	13.41	11.57	20.93	14.24
	Z	14.21	26.67	18.47	14.06	25.56	12.73
3	X	19.84	18.75	22.62	11.51	21.84	14.81
	Y	9.22	10.08	15.66	11.49	17.62	13.29
	Z	21.94	24.27	25.09	12.72	23.34	13.96
4	X	16.04	22.23	22.51	19.56	25.61	23.83
	Y	11.55	14.40	16.29	13.65	21.30	15.93
	Z	17.67	23.87	29.99	20.61	28.39	22.16
5	X	19.45	18.62	18.34	19	18.72	9.04
	Y	16.38	16.42	15.24	18.50	17.15	16.42
	Z	21.97	26.65	21.55	22	23.40	16.02

**Table 3:** Mean and Standard deviation (SD) of error between the gait parameters estimated from proposed and motion capture system at a walking speed of 0.28 m/s. E[SL] and E[ST] represents the error in step length and stride time respectively.

Subject	Foot	E[SL] (mm)		E[ST] (ms)	
		Mean	SD	Mean	SD
1	left	−30.95	11.59	0.36	10.7
	right	4.02	13.95	0.18	9
2	left	−26.42	8.36	0.23	20
	right	18.63	25.46	−0.9	26.4
3	left	−19.95	16.61	−1.1	26.9
	right	4.11	10.72	0.81	11
4	left	−17.74	11.98	0.00	16.9
	right	9.96	13.26	0.49	16.9
5	left	−16.21	21.39	1.2	24.2
	right	10.51	18.21	−0.59	26.5

**Table 4:** Mean and Standard deviation(SD) of error between the gait parameters estimated from proposed and motion capture system at a walking speed of 0.83 m/s. E[SL] and E[ST] represents the error in step length and stride time respectively.

Subject	Foot	E[SL] (mm)		E[ST] (ms)	
		Mean	SD	Mean	SD
1	left	−60.34	15.30	−0.43	14
	right	8.73	20.71	0.00	9.7
2	left	−62.27	17.76	0.19	10.7
	right	7.00	9.32	−0.37	8.5
3	left	−48.17	33.17	1.9	41.1
	right	−6.00	21.64	−0.2	19.9
4	left	−49.99	29.53	0.00	43.4
	right	6.09	21.28	0.38	18.7
5	left	−37.27	21.85	0.21	30.9
	right	11.71	13.55	−0.40	12.5

## Abbreviations

The following abbreviations are used in this manuscript:

3D	Three Dimensional
NLOS	Non-Line of Sight
LOS	Line of Sight
RMSE	Root Mean Square Error
PCC	Pearson’s Correlation Coefficient
UKF	Unscented Kalman Filter
URTS	Unscented Raunch-Tung Striebel Smoother
IC	Integrated Circuit
MAE	Mean Absolute Error
RF	Radio Frequency

## Appendix A. Steps for URTS

The procedure for computing smoothing distribution of step k from that of step *k* + 1 is explained here.

- Calculate the matrix of sigma points
  (A1) $$\chi_{k}=\left[m_{k}\;\ldots \;m_{k}\right]+\sqrt{\left(n+\lambda \right)}\left[0\;\sqrt{P_{k}}\;-\sqrt{P_{k}}\right]$$
- Transmit the sigma points through the dynamic model
  (A2) $${\hat{\chi }}_{k+1,i}=f\left(\chi_{k,i}\right),\;i=1,\ldots ,2n+1$$
- Predict the mean $m_{k+1}^{-}$, covariance $P_{k+1}^{-}$ and cross-covariance $C_{k+1}$ using weight matrices $W_{i-1}$.
  (A3) $$\begin{array}{c} m_{k+1}^{-}=\sum_{i}W_{i-1}^{mean}{\hat{\chi }}_{k+1,i}\\ P_{k+1}^{-}=\sum_{i}W_{i-1}^{cov}\left({\hat{\chi }}_{k+1,i}-m_{k+1}^{-}\right){\left({\hat{\chi }}_{k+1,i}-m_{k+1}^{-}\right)}^{T}+Q_{k}\\ C_{k+1}=\sum_{i}W_{i-1}^{cov}\left(\chi_{k,i}-m_{k}\right){\left({\hat{\chi }}_{k+1,i}-m_{k+1}^{-}\right)}^{T} \end{array}$$
- Compute the smoother gain, $SG_{k}$ and final smoothed mean, $m_{k}^{s}$ and covariance $P_{k}^{s}$.
  (A4) $$\begin{array}{c} SG_{k}=C_{k+1}{\left[P_{k+1}^{-}\right]}^{-1}\\ m_{k}^{s}=m_{k}+SG_{k}\left(m_{k+1}^{s}-m_{k+1}^{-}\right)\\ P_{k}^{s}=P_{k}+SG_{k}\left(P_{k+1}^{s}-P_{k+1}^{-}\right)SG_{k}^{T} \end{array}$$

The smoothing and filtering distribution of the last time step are same. Hence, $m_{T}^{s}=m_{T}$ and $P_{T}^{s}=P_{T}$.
